# Harnessing Plant Bioactive Compounds in Biomaterial Scaffolds for Advanced Wound Healing: A Comprehensive Review

**DOI:** 10.3390/biomedicines13102414

**Published:** 2025-10-02

**Authors:** Nur Syazana Sabarudin, Norshazliza Ab Ghani, Nazeha Ahmat, Eka Wahyuni Harlin, Looi Qi Hao, Juni Handajani, Fatimah Mohd Nor, Nur Izzah Md Fadilah, Manira Maarof, Mh Busra Fauzi

**Affiliations:** 1 Department of Tissue Engineering and Regenerative Medicine, Faculty of Medicine, Universiti Kebangsaan Malaysia, Jalan Yaacob Latif, Cheras, Kuala Lumpur 56000, Malaysia; syazana@ukm.edu.my (N.S.S.); norshazlizaabghani@gmail.com (N.A.G.); nazehaahmat@ukm.edu.my (N.A.); ekaharlin@gmail.com (E.W.H.); izzahfadilah@ukm.edu.my (N.I.M.F.); manira@ukm.edu.my (M.M.); 2 Advance Bioactive Materials-Cells UKM Research Group, Universiti Kebangsaan Malaysia, Bangi 43600, Malaysia; 3 My Cytohealth Sdn. Bhd., 5th Floor, Plaza Hamodal, Lot No. 15, Jalan 13/2, Section 13, Petaling Jaya, Selangor 46200, Malaysia; dr.daniellooi@cytoholdings.com; 4 Department of Oral Biology, Faculty of Dentistry, Universitas Gadjah Mada, Yogyakarta 55281, Indonesia; junihandajani@ugm.ac.id; 5 KPJ Ampang Puteri Specialist Hospital, Suite No. 3-13, No. 1, Jalan Mamanda 9, Taman Dato’ Ahmad Razali, Ampang 68000, Malaysia; drfatimahnor@gmail.com

**Keywords:** wound healing, plant bioactives, biomaterial-based drug delivery, regenerative medicine, phytochemicals, 3D bio-printed scaffolds

## Abstract

Wound healing remains a significant clinical challenge due to antibiotic-resistant pathogens, persistent inflammation, oxidative stress, and impaired tissue regeneration. Conventional therapies are often inadequate, necessitating alternative strategies. Plant bioactive compounds, including flavonoids, tannins, terpenoids, and alkaloids, offer antimicrobial, anti-inflammatory, antioxidant, and pro-angiogenic properties that directly address these challenges in wound healing therapy. However, their poor solubility, instability, and rapid degradation at the wound site limit clinical translation. Biomaterial-based scaffolds such as hydrogels, electrospun nanofibers, lyophilized dressings, and 3D-bioprinted constructs have emerged as promising delivery platforms to enhance bioavailability, stability, and sustained release of bioactive compounds while providing structural support for cell adhesion, proliferation, and tissue repair. This review was conducted through a structured literature search using PubMed, Scopus, and Web of Science databases, covering studies published between 1998 and 2025, with keywords including wound healing, phytochemicals, plant bioactive compounds, scaffolds, hydrogels, electrospinning, and 3D bioprinting. The findings highlight how incorporation of plant bioactive compounds onto scaffolds can combat resistant microbial infections, mitigate oxidative stress, promote angiogenesis, and accelerate tissue regeneration. Despite these promising outcomes, further optimization of scaffold design, standardization of bioactive formulations, and translational studies are needed to bridge laboratory research with clinical applications for next generation wound healing therapies.

## 1. Introduction

Skin is a multilayer organ in human bodies that serves as a tough and pliable barrier to protect the internal organs against the environment, while maintaining the dynamic homeostasis of the internal environment through the control of body fluid, thermoregulation, gaseous exchange, and immunological functions. Being the largest organ, skin is most vulnerable to various lesions that are caused intrinsically and extrinsically, such as harsh weather, contact with abrasive chemicals, genetic disorder, skin microbiome imbalance, and physical trauma [1]. Physical trauma may occur by accident or due to a surgical procedure, diseases, or infections. The injury of this barrier will create an opening for bacterial infiltration, hence disrupting the microbiome balance of the stratum corneum and resulting in unwanted microbial bioburden on the wound area. Understanding wound classification is crucial, as it enables accurate assessment, helps the selection of appropriate therapeutic strategies, and anticipate potential challenges during the treatment.

In response to a wound, the body initiates a series of complex biological processes aimed at restoring tissue integrity. Wound healing process will take place in four sequential and coinciding stages, namely hemostasis, inflammation, proliferation, and tissue remodeling [2]. For optimal tissue restoration, the four stages of wound healing must occur in their programmed sequence, each within a specific and timely duration. Any slight interruptions, anomalies, or extensions to the wound healing process could prolong or cause an unhealed wound [3].

Given the limitations of the body’s natural healing capacity, the management of wounds has long posed a critical challenge in medical practice, dating back to ancient times. One of the earliest documented accounts of wound treatment dates to 2000 B.C., found on a Sumerian clay tablet that outlines ‘three healing gestures,’ essentially the basic principles of wound care [4]. Traditional therapies mainly incorporate natural compounds derived from various plants and animals, whose application is not only limited to wound therapeutics, but also treatment of various diseases and complications. From approximately 270,000 species of discovered plants, about 50,000 species were reported as traditional medicine for various treatments in various forms [5].

Natural products derived from medicinal plants provide abundant sources of biologically active compounds, many of which have inspired pharmaceutical development. Current challenges in wound healing, including antibiotic-resistant infections, prolonged inflammation, and impaired tissue regeneration, highlight the urgent need for effective alternatives. Plant-derived bioactive compounds are especially attractive due to their biocompatibility, sustainability, availability, and cost-effectiveness. Their antioxidative, antimicrobial, anti-inflammatory, and pro-angiogenic activities make them promising candidates to address multiple challenges in wound treatment [6]. However, despite the identification of approximately 500,000 plant species worldwide, only about 1% have been phytochemically investigated [7]. To maximize their therapeutic potential, recent advances in biomaterial science have focused on integrating these bioactive compounds with engineered scaffolds. The emerging field of biomaterials has enabled the development of extracellular matrix-mimicking scaffolds loaded with plant bioactive compounds, serving as controlled-release drug carriers to enhance wound repair [8].

This review discusses the therapeutic properties of plant bioactive compounds, the strategies of incorporating them with biomaterial scaffold, their mechanism of action, fabrication method and associated challenges. The future perspectives in using plant bioactive compounds for the treatment of wounds are also discussed.

## 2. Physiology of Wound Healing

**Figure 1:**
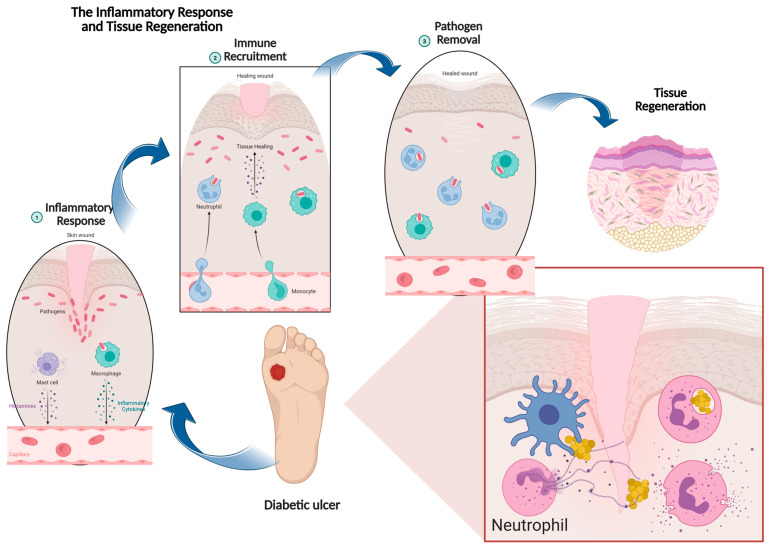
The sequential process of chronic wound healing process in diabetic ulcer, beginning with inflammatory response and immune cell recruitment, progressing to pathogen removal, and culminating in tissue regeneration. Image created by using biorender.com.

In wound healing process, many cellular and humoral factors will come into play, such as enzymes, cytokines, growth factors, proteins, and hormones [9]. Once a wound occurs, hemostasis will start within an hour, initiated through vasoconstriction of the blood vessel wall [10]. During primary hemostasis, aggregation of platelets occurs to form a platelet plug. At the same time, concurrently soluble fibrinogen is configured to make up a fibrin mesh via activation of coagulation complexes in a process called secondary hemostasis, purposely to stop bleeding [10,11]. Simultaneously, the inflammation phase occurs through the mediation of immune cells, and the neutrophils and monocytes are recruited to the wound site to perform phagocytosis to protect against bacterial invasion and clear off cell debris [12]. The role of macrophages in wound healing is crucial, as their absence during the inflammatory phase was found to reduce epithelial repair and tissue granulation, resulting in delayed healing [13]. The sequential stages of wound healing, particularly in chronic conditions such as diabetic ulcer are illustrated in Figure 1.

Initially, the inflammatory response is triggered by the presence of pathogens, activating neutrophils and macrophages that release signaling molecules to recruit additional immune cells. These cells contribute to pathogen clearance, as shown in the pathogen removal stage, where neutrophils phagocytose microbes and release antimicrobial agents. Once the infection is controlled, the wound environment shifts toward resolution, enabling fibroblasts and other reparative cells to rebuild the extracellular matrix and restore structural integrity. Ultimately, this coordinated sequence leads to tissue regeneration, reflected in the reformation of healthy skin layers, highlighting how effective immune regulation is pivotal in overcoming chronic wound complications such as those seen in diabetic ulcers. When inflammation starts to decline, the proliferation phase takes place. The proliferation phase is characterized by tissue granulation, epithelial regeneration, and neovascularization activities. Tissue granulation is driven by the fibroblast, forming a constitution of collagen, proteoglycans, granulocytes, and angiogenic growth factors which compose the wound matrix, contributing to its stabilization [14]. Following that, reepithelialization commences as keratinocytes proliferate and migrate from the edge of the wound or epithelial stem cells from nearby hair follicles and sweat glands, released by the collagenase and elastase [15]. The nitric oxide-releasing macrophage, wound-related cytokines, and growth factors are responsible for activating reepithelialization [16]. Next, the neovascularization process takes place when vascular endothelial growth factor (VEGF), platelet-derived growth factor (PDGF), basic fibroblast growth factor (bFGF), and the serine protease thrombin trigger the activation of endothelial cells to form new blood vessels from the remaining vessels. Otherwise, new vessels descended from endothelial progenitor cells (EPCs) derived from bone marrow through vasculogenesis [17].

Remodeling is the final stage of wound healing, usually commencing after approximately three weeks post-injury. At this stage, the tissue granulation process matures, which is characterized by the decrease in the number of blood capillaries as they diffuse into larger vessels, glycosaminoglycans, and the occurrence of wound contracture due to the expression of smooth muscle action by myofibroblasts [18]. Additionally, the type and organization of collagen deposition gradually change when collagen type I synthesis is increased to replace the collagen type III produced in an earlier stage of wound healing. This contributes to the higher tensile strength of the tissue ECM [19]. However, the newly restored tissue will never gain its original pre-wounded tensile strength; only 80% of the maximum tensile strength will be recovered [20]. Orientation and the structural composition of the collagen fiber that make up the wound ECM determine the rate of scarring [21]. Complications such as hypertrophic scarring, keloids, or non-healing ulcers underscore the limitations of natural repair mechanisms and highlight the need for advanced therapeutic interventions, including biomaterial-based scaffolds loaded with bioactive compounds, to support effective wound healing.

## 3. Pro-Healing Plant Bioactive Compounds

### 3.1. Polyphenol

Polyphenols that have a wide range of complex structures are a large family of naturally occurring phenols that have been studied worldwide. The basic monomer in polyphenols is phenolic rings, and the strength of the rings could classify the polyphenols into many classes, such as phenolic acids, flavonoids, phenolic alcohols, and many more [22]. Besides that, there is a study that shows that some classification of polyphenolic groups based on phenol rings varies according to the slightly different interpretation of the researcher. However, the basic classification of polyphenols is agreed upon in five classes: flavonoids, phenolic acids, stilbenes, lignans, and others [23]. Flavonoids that are giving roles in flavor, color and pharmacological activities; due to its immunomodulatory, anti-inflammatory and anticancer activities may be extracted through grapes skin, *Morus alba*, *Platycladus orientalis* leaves, *Gordonia axillaris,* tomato, *Morus nigra* leaves, and *Radix Astragal* roots [24]. Flavonoids are said to promote the formation of new epithelium by enhancing keratinocyte migration and epithelial attachment to wounds, thereby releasing growth factors that stimulate mitosis and epithelial hyperplasia, advancing the proliferative phase of healing. Besides that, it is also able to increase the levels of SOD, CAT, GSH, GST and GPx and combating inflammation, hypertension and dementia [25].

### 3.2. Terpenoids

Other than the polyphenol group, terpenoids also attract researchers’ attention for their pharmacological effects. Terpenoids, also referred to as isoprenoids, are among the most abundant and structurally diverse natural products in plants. There are a few types of terpenoids that exist, such as hemiterpenoids, monoterpenoids, iridoids, sesquiterpenoids, diterpenoids, sesterterpenoids, triterpenoids, tetraterpenoids, polyterpenoids, and irregular terpenoids. These metabolites may be found in plants such as galbanum (*Ferula gummosa*), hyssop, Apocynaceae, Gentianaceae, and many more [26]. In wound management, terpenoids that exhibit antimicrobial activity have potential as wound dressing when combined with halloysite nanocomposites [27]. Besides playing a role as an antimicrobial agent, iridoid compounds found in the *Scrophularia* genus are said to have proven biological activities such as anti-inflammatory, anticancer, and antiprotozoal [28]. This study is supported by another study conducted, [29] where the iridoid glycoside extract of *Lamiophlomis rotata* (Benth.) Kudo may expedite the wound healing processes by inducing polarization of M2 macrophages.

### 3.3. Alkaloid

Another group of naturally occurring chemical compounds from plants is alkaloids, which contain mostly basic nitrogen atoms. This nitrogen atom in the alkaloid gives the compound drug its properties. Alkaloids have been classified into a few categories, which include piperidine, tropane, purine, pyrrolizidine, imidazole, quinolizidine, isoquinoline, and pyrrolidine alkaloids [30]. In plants themselves, alkaloid helps as growth regulators and substitutes for minerals, while in humans, alkaloid is commonly used in the pharmaceutical industry as antimicrobials, anti-HIV, and anti-parasitic [31]. In 2021, [32] proven that harmala alkaloid-rich fraction (HARF) loaded on nanoparticles obtained 94.4 ± 8.0% wound closure percentage compared to the unloaded HARF and blank nanoparticles and displayed potential wound healing properties via antibacterial activity. In addition, alkaloid also promotes cell migration and reduces the cell gaps, which helps expedite wound healing. However, due to its drug properties, the toxicity of the alkaloid for wound treatment should be considered [33].

## 4. Properties of Plant Bioactive Compounds for Wound Healing and Their Mechanism

### 4.1. Antimicrobial Properties

**Figure 2:**
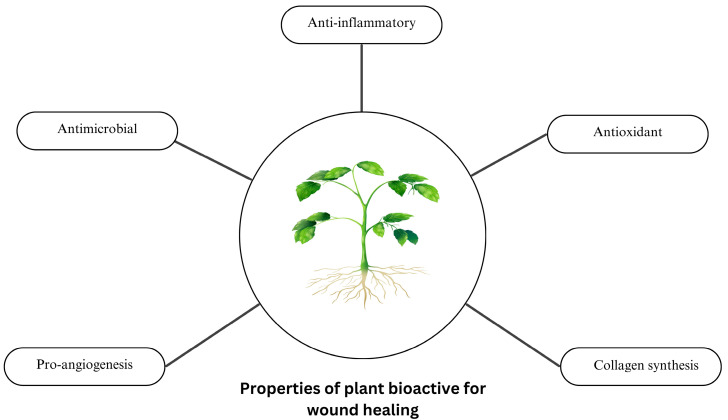
Overview of the essential properties of plant-derived bioactive compounds that contribute to various stages of wound healing.

Many phytocompounds have been extensively investigated for their therapeutic roles in different phases of wound healing (Figure 2). These bioactive are used as a stand-alone isolated compound or a mixture of whole plant extracts to treat open wounds. One of the challenges in wound management is wound contamination by local microbial bioburden. Usage of natural bioactive as antimicrobial wound treatment has shed some light on curbing this issue since the use of antibiotics can potentially lead to antibiotic-resistant infections [34]. Wound infections with antibiotic-resistant pathogens contribute to increased risk of mortality, morbidity, and overall cost for treatment [35]. Although it is not possible to create a sterile wound environment devoid of bacteria, it is crucial to provide a host-manageable bioburden environment favorable for healing.

The antibacterial application of plant phytochemicals for wound therapy has been well documented. The antiseptic properties are derived from plant lipophilic alkaloids and essential oils. They generally have low solubility in polar solvents; hence, they are best formulated in oil-in-water suspension to ensure their stability [36]. Incorporation of plant bioactive compounds into wound dressing has proven to enhance the progression of wound healing. Essential oils derived from rosemary and oregano present an inhibitory effect against biofilm-forming bacteria when incorporated in cellulose acetate fibers wound dressing [37]. Similarly, hydrogel film infused with natural flavonoids and polyphenols presents enhanced in vitro bactericidal activities [38]. In addition, an in vivo study indicated that quercetin has antimicrobial properties to protect the injury site from bacterial inhibition, thus maintaining a favorable ECM for cell proliferation and differentiation toward wound closure [39].

The antibacterial effect of natural compounds acts by attaching to the surface of the cell to penetrate the phospholipid bilayer of the bacterial cell membrane. This action will disturb the membrane permeability and disrupt normal cell physiology, eventually causing cell death. The bactericidal mechanisms of plant metabolites are not specific and vary, thus beneficial to protect against the emergence of bacterial resistance. Flavonoids possess a wide range of bactericidal mechanisms, from interference with nucleic acid synthesis, metabolism of energy, impeding cell membrane porins, cell attachment and development of biofilm, and disrupting membrane permeability, as well as attenuation of bacterial virulence [40]. Meanwhile, tannins’ antimicrobial activities are contributed by their astringent effect due to their molecular structure. Tannins disrupt the gene expression of bacterial pathogenicity, interfering with fatty acid biosynthetic pathways, act as iron chelating agents, and inhibit bacterial wall synthesis and disrupt the membrane [41]. Alkaloids also have different mechanisms of action against microorganisms, including nucleic acid synthesis inhibition, respiratory and enzyme inhibition, disrupting bacterial membranes, and inhibiting virulence factor encoding genes [42]. Terpenoids exert a wide spectrum of bactericidal activities via inhibiting essential molecular synthesis pathways, including nucleic acid, proteins, disruption of cell wall components, and interference with DNA replication and bacterial metabolisms [43,44].

Recently, the synergistic influence of combined plant phytoextract with antibiotics has been proposed as an option to fight against emerging microbial resistance, as using monotype herbal antiseptics may not eliminate the chance of resistance [45]. Plant methanolic and ethyl acetate extracts were proven to have synergistic capacity and an antibiotic adjuvant effect in treating bacterial resistance [46]. A combination of plant extracts provides pronounced pharmacological benefits through synergism, enhanced therapeutic effectiveness, while reducing the minimum effective dose to minimize cytotoxicity. Despite this effect may be true at some point, further study is required to understand the underlying key mechanisms.

### 4.2. Antioxidant Activity

Upon injury, multiple cascade of healing processes takes place, and these processes utilize oxygen to synthesize ATP, which is the main source of energy. Consequently, the concentration of reactive oxygen species (ROS) increases, and the wound site is a byproduct of the ATP required healing process. An increase in ROS is associated with the formation of chronic inflammation, impairing proper healing. Nevertheless, the body requires a balanced level of ROS in order to keep normal body functions, which facilitate wound healing, as ROS is known to be directly involved in haemostasis, re-epithelialization, and angiogenesis. Excessive presence of ROS impairs proper healing. Hence, the body possesses an antioxidant defense system that can detect abnormal oxidant levels and subsequently react accordingly to restore the balance of the ecosystem.

Antioxidant is a substance that functions to control the oxidation of biomolecules via interaction with reactive species such as ROS and RNS, and acts as an oxidative stress-related chelating agent, such as metal ions and enzyme modulator [47]. Free radicals are produced at the site of injury as a byproduct of the healing process, and the presence of an antioxidant will alleviate the damage caused by these free radicals to surrounding cells [48]. Plant phenolic compounds largely contributed to the plant antioxidant activities. These compounds include phenolic acids, flavonoids, tannins, lignans, and stilbenes, which act on different wound healing phases to limit cellular damage. Flavonoid affects the prostaglandins and macrophages during the wound inflammatory phase to control cellular damage [49]. Besides that, flavonoids are also associated with the durability of collagen fibers to aid in wound contraction and re-epithelization [50]. Tannin is a naturally synthesized plant metabolic product widely studied for its wound healing potential. The antioxidant effect of tannins promotes wound closure via scavenging action against ROS and free radicals, upregulates the formation of new capillaries, and the proliferation of fibroblasts [51].

Vitamin E, a nonenzymatic plant antioxidant, is known to protect cell membranes against lipid peroxidation via direct neutralization of peroxyl radicals, superoxide, and singlet oxygen. In contrast, vitamin C boosts this antioxidant effect by salvaging the tocopherol radical, escalating Vitamin E’s effectiveness, and producing dehydroascorbic acid to help further reduce oxidative stress. Other plant-derived compounds, such as genistein, are also widely studied for their antioxidant activities. In the diabetic wound model, genistein alleviates oxidative stress by suppressing inducible nitric oxide synthase (iNOS), forkhead box O transcription factor (FoxO1) activities, as well as modulation of glutathione level and inhibition of lipid peroxide formation [52,53,54]. Protection against free radicals, the role of plant-derived antioxidant enzymes in mitigating oxidative stress, has also been studied. Superoxide dismutase (SOD) facilitates the breakdown of superoxide radicals into hydrogen peroxide and then converted to water and oxygen [55]. Application of SOD-loaded wound dressing showed a promising effect on the healing of chronic wounds by accelerating epidermal re-epithelialization and collagen deposition [56].

### 4.3. Anti-Inflammatory Effects

Plant-derived metabolites are known for their potent anti-inflammatory properties to support wound healing. Inflammation is required to protect the injured cells against microbial attack, physical injury, and defective immunity. However, above a certain extent, prolonged inflammation can cause a non-healing wound. Inflammation can be either acute or chronic, both of which play a significant role in maintaining proper healing. Anti-inflammatory effects from plant metabolites have been studied to modulate the inflammatory response for the treatment of chronic wounds and accelerate healing. Plant-derived flavonoids, tannins, terpenoids, and alkaloids are a few of the well-studied metabolites for the control of wound inflammation. Flavonoids, the largest group of plant phenols, are extensively known for their anti-inflammatory properties, and their application in wound therapy has been investigated in vitro and in vivo. Flavonoids regulates inflammatory response by acting on multiple signaling molecules, like NF-κB, iNOS, cytokines, cyclooxygenase (COX), and matrix metalloproteinases (MMPs) [57].

Wound sites are vulnerable to contamination and consequently alter the surrounding microenvironment, which leads to downregulation of pro-inflammatory cytokines, excess lactate, pH imbalance, toxin release, and increased ROS molecules [58]. These adverse environments discourage re-epithelialization, regulation of growth factors, and other pro-healing extracellular matrix components. Application of flavonoids, triterpenoids, and tannins has been shown to improve wound microenvironment through multi-action, such as anti-microbial and anti-inflammatory. While its anti-microbial capacity protects the wound from microbial contaminants, these phytochemicals regulate the expression of IL-10 while suppressing the spike of TNF and IL-6 expression. IL-10 helps to reduce the expression of pro-inflammatory and profibrotic mediators. This results in a reduction in inflammatory cell infiltration at the wound site. The presence of TNF inhibits the production of collagen and hydroxyproline, which are both crucial for the regenerative phase of wound healing [59].

**Figure 3:**
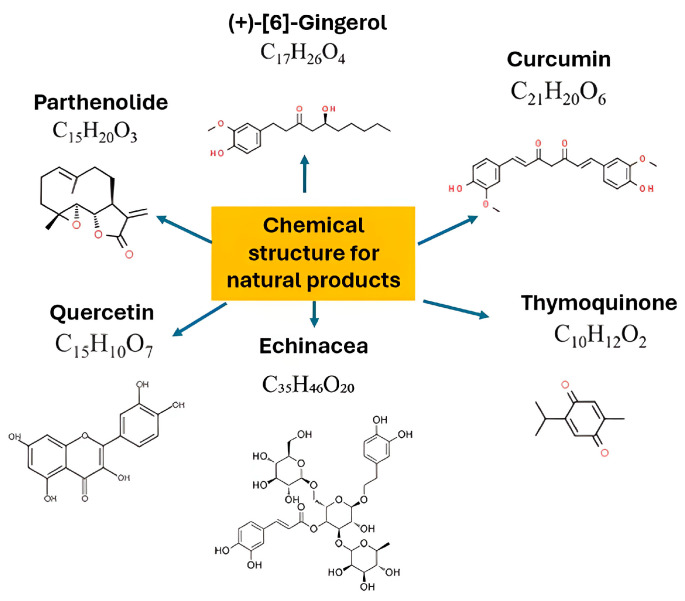
Representative chemical structures of selected plant-derived compounds with reported anti-inflammatory potential, including sesquiterpene lactones (parthenolide), phenolics (curcumin, [6]-gingerol), flavonoids (quercetin), phenolic acid derivatives (caffeic acid derivatives from *Echinacea*), and quinones (thymoquinone). Image created by using biorender.com.

To further illustrate the diversity of anti-inflammatory phytochemicals, Figure 3 presents representative chemical structures of well-studied natural compounds. For instance, parthenolide, a sesquiterpene lactone from *Tanacetum parthenium* (feverfew), inhibits NF-κB activation and reduces IL-6 and TNF-α secretion, whereas [6]-gingerol from ginger suppresses prostaglandin and cytokine production. Curcumin, a polyphenolic compound from turmeric, interferes with MAPK and NF-κB pathways to attenuate TNF-α, IL-1, and IL-6 release. Quercetin, a flavonoid abundant in fruits and vegetables, reduces oxidative stress and enhances IL-10 production. Similarly, caffeic acid derivatives and polysaccharides in *Echinacea* regulate immune cell activity and reduce pro-inflammatory mediator synthesis, while thymoquinone from *Nigella sativa* demonstrates broad antioxidant and immunoregulatory effects through modulation of ROS and cytokine networks. Collectively, these compounds exemplify how structurally diverse phytochemicals converge on common inflammatory pathways, highlighting their promise as complementary agents in wound management and drug discovery.

### 4.4. Pro-Angiogenesis

During the proliferation phase of wound repair, new blood vessels are formed in a process called angiogenesis. In a healthy tissue, microvasculature maintains a state of homeostasis, where the supply of oxygen and nutrients is balanced with the removal of waste and carbon dioxide. However, in a state of injury, vascular microstructure dysfunction causes the accumulation of cellular fluid, inflammation, and the onset of hypoxia [60]. Hypoxic condition signals angiogenesis as they activate endothelial cells along with inflammatory cytokines to trigger the recruitment of immune cells. In a non-healing chronic wound, angiogenesis is reduced due to the presence of anti-angiogenic factors. Diabetic wounds pose a significant reduction in cell surface heparan sulfate proteoglycans that function in binding of growth factors to their respective receptors [61]. Accordingly, proteomic analysis of diabetic wound exudates indicates a high profile of anti-angiogenic proteins despite upregulation of angiogenic factors [62].

**Figure 4:**
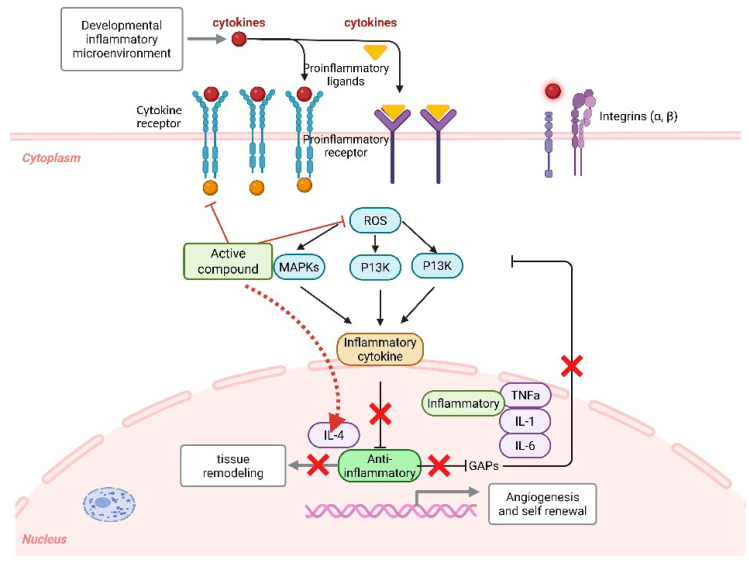
Natural compounds modulate inflammatory signaling pathways by suppressing pro-inflammatory cytokine production while enhancing anti-inflammatory mediators. Through the inhibition of MAPKs, PI3K, and ROS pathways, these active compounds reduce the secretion of TNF-α, IL-1, and IL-6 while promoting IL-4-driven anti-inflammatory responses and tissue remodeling. Image created by using biorender.com.

Many bioactive phytochemicals, including polyphenols, flavonoids, terpenoids, and polysaccharides, act as inhibitors of pro-inflammatory mediators such as TNF-α, IL-1β, and IL-6, which are critical drivers of chronic inflammatory diseases (Figure 4). By interfering with upstream regulators like MAPKs, PI3K/AKT, and ROS generation, natural products effectively reduce the activation of transcription factors such as NF-κB and AP-1, thereby limiting the transcription and secretion of pro-inflammatory cytokines. This downregulation not only suppresses excessive immune activation but also protects tissues from chronic damage associated with unresolved inflammation.

In addition to dampening pro-inflammatory signaling, natural products enhance the production and activity of anti-inflammatory cytokines, contributing to immune balance and tissue repair. Compounds such as curcumin, resveratrol, and fucoidan have been shown to stimulate IL-4 and IL-10 production, which are essential in shifting immune responses toward a regulatory and reparative phenotype. These cytokines promote tissue remodeling, angiogenesis regulation, and homeostatic repair processes that are critical for the resolution of inflammation. Overall, the dual action of natural products suppressing pathogenic inflammatory cytokines while enhancing protective anti-inflammatory mediators which represents a promising therapeutic strategy to restore immune homeostasis and support regeneration in chronic inflammatory conditions.

Bioactive extracted from various plants have been investigated for their angiogenic potential to promote the repair of nonhealing wounds. Their mechanisms of action vary but mostly target the proliferation phase of healing via the upregulation of vascular endothelial growth factor (VEGF). Asiaticoside has demonstrated pro-angiogenic activity by increasing the expression of VEGF in burn wound models. While other studies indicated asiaticoside promotes angiogenesis via stimulation of keratinocytes to produce monocyte chemoattractant protein-1 (MCP-1), subsequently leading to elevated VEGF-A levels [63,64]. Polyphenols, and in particular, flavonoids are proven to be angiogenesis promoters. Flavonoids enhance the expression of angiogenesis-related biomarkers to stimulate neovascularization at the wound site [65]. Study in a diabetic wound model using hesperidin, observed the angiogenic potential of this compound via upregulation of VEGF-c, Ang-1/Tie-2, TGF-, and Smad-2/3 mRNA. In addition to this, hesperidin’s antimicrobial properties alleviate the risk of infection, thereby supporting the epithelialization process for faster wound closure [66].

Ferulic acid improved wound healing by promoting faster epithelialization and increasing the content of important wound-healing components such as hydroxyproline and hexosamine [67]. Additionally, ferulic acid is associated with modulation of hypoxia-inducible factor-1 alpha (HIF-1α), further enhancing VEGF and PDGF production [68]. The common pro-angiogenic potential of plant extracts is mediated via activation of VEGF/VEGFR pathways. A study on ginsenoside, the active ingredient of *Panax notoginseng,* enhances angiogenesis in streptozotocin-induced diabetic rats by mediating miR-23a levels to inhibit the regulation of interferon regulatory factor-1 (IRF-1). Consequently, IRF-1 signals inducible nitric oxide synthase (iNOS) and a subsequent rise in nitric oxide (NO) production. This leads to an increase in VEGF levels, as well as enhanced cell proliferation, anti-apoptotic activity, and the migration capability of endothelial cells, all of which contribute to improved wound healing [69].

### 4.5. Collagen Synthesis

Plant bioactive compounds have long been known to promote collagen synthesis during the tissue repair process. These compounds have been demonstrated to enhance collagen biosynthesis and tissue regeneration, indicating their pro-healing potential. Polysaccharide-rich *Aloe vera* leaf gel extract encapsulated in liposomes has been shown to increase the synthesis of collagen by 23%. Meanwhile, treatment of *A. vera* in rats promotes collagen turnover via upregulation of urinary hydroxproline levels and lysyl oxidase activity [70]. Asiatic acid, a triterpenoid derived from *Centella asiatica*, enhances collagen production in fibroblasts, promotes the accumulation of extracellular matrix, and improves tensile strength of newly formed tissues during the proliferative and remodeling phases [71].

Flavonoids enhance collagen biosynthesis during tissue repair via multiple mechanisms. Primarily, flavonoids modulate Wnt/β-catenin, TGF-β, and MAPK/ERK pathways, which results in cellular proliferation and differentiation, thus improving the synthesis of collagen [72]. Antioxidant properties of the flavonoid protect newly formed collagen fibers from degradation effects from residual ROS, thus maintaining extracellular matrix integrity [73], while its anti-inflammatory activity impedes inflammatory cytokines against collagen production. Quercetin is a flavonoid known to upregulate the expression of collagen type III via modulation of SIRT3/TGF-β/Smad3 axis signaling pathways [74].

## 5. Role of Biomaterials as Delivery Systems in Wound Healing

The healthcare industry faces significant challenge due to tissue and organ failure caused by traumatic injury, diseases, and congenital conditions. Existing therapy includes procedures such as drug therapy, surgery, and artificial implantation. However, these procedures are curbed by many factors such as host tissue rejection, immunological status, graft quality, and limited availability of tissue donors. Due to these, tissue engineering strategies are considered an alternative therapy to address the shortcomings of the conventional approach. Scaffolds, in combination with cells and regulatory signals such as growth factors and cytokines, are considered a triad of tissue engineering. The ideal scaffold of engineered tissues must bio-mimic the target tissues’ extracellular matrix in terms of functional behavior and mechanical support [75]. Scaffold design is crucial to ensure effective restoration of the structural and functional integrity of the injured tissues. The ideal scaffold for wound therapy shall meet certain physical and mechanical characteristics and provide a physiological environment to support cell proliferation, cell adhesion, and differentiation. Besides that, the scaffold should exhibit high porosity, a large surface area to volume ratio, with interconnected structures, while giving enough flexibility to conform to the shape of the wound. Aside from being biocompatible and biodegradable, the scaffold should have a degradation rate that suits the wound healing duration. It should retain a moist environment, which favors cellular adhesion, growth, and migration, stimulating angiogenesis, accelerating granulation tissue formation, and supporting re-epithelialization [76].

As a critical component in tissue engineering, various types of scaffolds have been developed to meet the requirements of tissue engineering (Table 1). Scaffolds can be categorized according to their material composition and fabrication techniques. Material-based scaffolds can be made of natural polymers, synthetic polymers, or a combination of both.

Conventionally, scaffolds are fabricated using techniques like solvent casting, freeze-drying, and electrospinning. Solvent casting involves dissolving polymers in a solvent, then casting the mixture in molds, followed by the removal of the solvent to leave behind a solid scaffold. This method allows for controlled porosity by using porogens, though it often results in limited control over pore size and architecture. Freeze-drying method, or lyophilization, creates highly porous structures by freezing a polymer solution and sublimating the solvent to produce scaffolds with interconnected geometry ideal for soft tissue engineering [84]. However, this method limits the precision required for more complex tissue structures. Electrospinning allows the creation of fibrous scaffolds that closely mimic tissue ECM by using electric fields to draw polymer fibers into thin strands. While electrospun scaffolds closely resemble native tissue environments, particularly in nerve and vascular tissue engineering, the random fiber deposition can result in poor control over scaffold architecture [85].

In addition, more advanced scaffolds are produced using 3D printing and bioprinting, which allow accurate control over scaffold mechanical properties to better suit cell attachment and tissue compatibility. Three-dimensional bioprinting utilizes layer-by-layer deposition of biomaterials to create scaffolds with complex geometries tailored to specific needs [86]. This technique allows for the customization of pore sizes, which can enhance nutrient diffusion and promote cell attachment, leading to better tissue integration. Moreover, incorporating living cells into the printing process allows for the construction of tissue-like structures that not only support cells but also mimic the native tissue environment more accurately. This high level of control over scaffold architecture is crucial for applications such as bone regeneration and organ biofabrication, where precise tissue compatibility and mechanical strength are critical [87]. In summary, scaffold design in wound therapy should not be viewed simply as a structural requirement but as a multifunctional platform for the delivery of plant bioactives. By combining appropriate biomaterials with tailored fabrication strategies, scaffolds can be engineered to accelerate wound healing while harnessing the therapeutic potential of phytochemicals.

## 6. Strategies for Incorporating Plant Bioactive Compounds into Various Scaffolds

**Figure 5:**
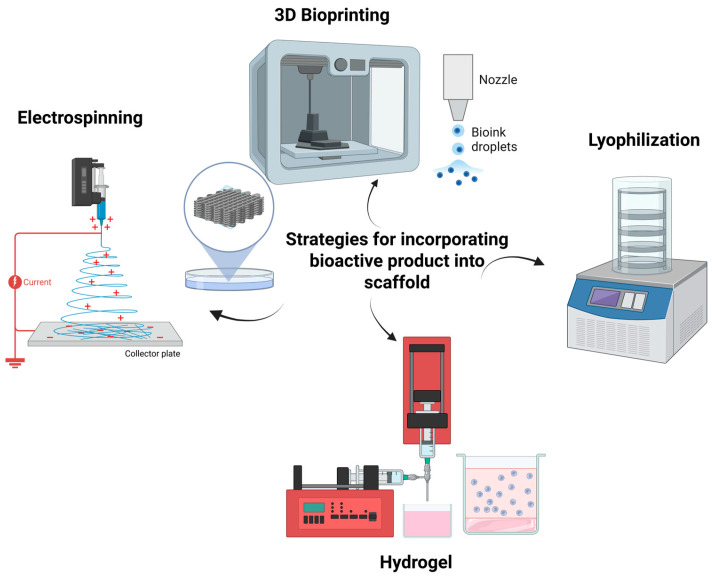
Common approach to incorporate plant bioactives into the scaffold. Image created using biorender.com.

Incorporating various pro-healing plant bioactives onto biomaterial scaffolds has been a prime focus to promote tissue regeneration at the targeted site. Bioactive-loaded scaffolds provide an added advantage over traditional scaffolds by stabilizing the delivery of the medications on the wound site, providing sustained and prolonged release of bioactive, giving optimal bioavailability of the bioactive, ensuring the delivery of the compound reaches its targeted site, and making sure their maximum effect lasts throughout the therapeutic process [88]. The incorporation strategies of plant bioactive compounds into the scaffold were illustrated in Figure 5 and the summary of different strategies is tabulated in Table 2.

### 6.1. Electrospinning

Electrospinning is a fabrication technique that utilizes electrostatic forces to turn biocompatible polymer into a fibrous scaffold [89]. The advantage of fibrous scaffolds produced via electrospinning lies in their nanoscale and microscale interlocking pores, which mimic tissue extracellular matrix (ECM), thus promoting a moist environment for cell proliferation and tissue regeneration. High surface area provided by the electrospun fiber allows for cell attachment, proliferation, and tissue regeneration, while embedded phytochemicals offer therapeutic benefits to accelerate the overall healing process.

The fabrication of plant bioactive compounds into scaffolds via the electrospinning method has been reported using various plant extracts. Fabrication of Grape seed extract-loaded silk fibroin nanofibrous mats was achieved by green electrospinning, which results in sustained release of bioactive via diffusion and demonstrates superior antioxidant activity [90]. Electrospun scaffolds made from polylactide (PLA) and enhanced with extracts from *Portulaca oleracea* have demonstrated the ability for sustained release of therapeutic bioactive molecules that promote cell proliferation and tissue regeneration [91]. The use of phytochemicals such as flavonoids, tannins, and terpenoids in electrospun scaffolds has been shown to optimize their antimicrobial properties. Incorporation of *A. vera* extract into electrospun nanofibers has been reported to provide significant antibacterial and anti-inflammatory effects, which are beneficial for preventing infections in wound sites [92].

**Table 2:** Summary of different strategies employed to incorporate plant-derived bioactive compounds into scaffolds for wound healing.

Fabrication Method	Description	Incorporated Plant Bioactives	Healing Benefits	References
Electrospinning	Uses electrostatic forces to produce nanofibrous scaffolds with ECM-like pores.	Grape seed extract, *P. oleracea*, *A. vera*, flavonoids, tannins, terpenoids.	High surface area for cell attachment and proliferation; sustained release of bioactives; antioxidant, antimicrobial, anti-inflammatory activity; tunable mechanical properties; portable in situ electrospinning enables direct wound dressing.	[91,92,93,94,95,96,97]
3D Bioprinting	Precise fabrication of customized scaffolds mimicking tissue architecture.	Flavonoids, polyphenols, essential oils (in alginate/gelatin bioinks).	Allows spatial control of cells and bioactives; customizable scaffold shape; enhanced antimicrobial, anti-inflammatory, and angiogenic activity; gradient delivery of compounds improves diabetic wound healing.	[98,99,100]
Hydrogel Formulation	Hydrophilic polymer networks (90% water) with high elasticity and moisture retention.	Cocoa extract, plantain peel, *A. vera, Calendula officinalis*, curcumin, *Kunzea ericoides*.	Maintains moist environment; absorbs exudates; supports keratinocyte migration and fibroblast proliferation; controlled degradation; sustained release of bioactives; enhanced antibacterial, antioxidant, immunoregulatory effects.	[101,102,103,104,105,106,107,108,109]
Lyophilization	Freeze-drying at low temperature to preserve phytochemicals and create porous scaffolds.	*Croton oblongifolius, Spinacia oleracea, Cissus quadrangularis*	Produces highly porous scaffolds (>90% porosity); excellent fluid absorption; stable phytochemicals; sustained release; improved biocompatibility, antioxidant, antibacterial, and bone tissue repair properties.	[110,111,112,113]

Moreover, the mechanical properties of electrospun scaffolds can be tailored by selecting appropriate polymer blends. For instance, combining polyurethane and gelatin in electrospun scaffolds has created a structure closely resembling the natural ECM, promoting faster cell infiltration and granulation tissue formation [93]. This is crucial for accelerating the wound healing process, as a well-structured scaffold can facilitate the migration of fibroblasts and other essential cells to the wound site [94]. The emergence of the in situ electrospinning technique allows for direct deposition of nanofibers, hence ensuring its compatibility and efficiency to help tissue repair. A portable electrospinning device in combination with thymol-loaded ethanol-soluble polyurethane has allowed on-site wound dressing application that conforms to wound shapes while providing optimum dressing with add-on antibacterial bioactive for enhanced healing [95].

Despite the advantages, electrospinning strategies posed quite a challenge during translation of the plant bioactives-loaded scaffold for clinical applications. This is because, this technique requires volatile organic solvents from the spinning process which may become entrapped within the fibers and subsequently leach out along with the incorporated drug leaving a potentially toxic residue [96]. Furthermore, while electrospinning is relatively cost-effective at the laboratory scale, scalability and manufacturing feasibility remain limited. Producing uniform nanofibers in large batches is challenging, with difficulties in controlling pore size and fiber alignment at an industrial scale [97]. The requirement for specialized equipment, solvent recovery systems, and strict process control further increases production costs. These issues underscore the need for ongoing research into greener solvents, continuous electrospinning systems, and scalable manufacturing strategies to translate phytochemical-loaded electrospun scaffolds from bench to bedside.

### 6.2. 3D-Bioprinting

The application of 3D bioprinting incorporated with plant bioactive compounds for wound healing is an innovative approach that leverages the advantages of both bioprinting technology and the therapeutic properties of natural compounds. This method allows for the precise fabrication of scaffolds that can mimic the complex architecture of human tissues while delivering bioactive substances that promote healing. Three-dimensional bioprinting enables the creation of customized scaffolds tailored to the specific shape and size of a wound, which is crucial for effective healing. Incorporating plant bioactive compounds, such as flavonoids, polyphenols, and essential oils, into these scaffolds can enhance their biological properties, including antimicrobial activity, anti-inflammatory effects, and promotion of angiogenesis [98,99]. For instance, bioinks derived from natural sources, such as alginate or gelatin, can be infused with plant extracts known for their wound-healing properties, creating a scaffold that not only provides structural support but also actively participates in the healing process [98].

One significant advantage of 3D bioprinting is its precise spatial control over the distribution of cells and bioactive compounds to cover the wound area. This precision allows for the creation of gradients of bioactive agents, which can be strategically placed to enhance cellular responses at the wound site. For example, a study demonstrated that scaffolds printed with a gradient of growth factors and plant extracts could significantly improve the healing of diabetic wounds by promoting cell migration and proliferation [100]. The ability to create such gradients is particularly beneficial in wound healing, where different phases of healing may require varying concentrations of bioactive compounds.

Nevertheless, this approach is not without processing and translational challenges. Many phytochemicals are sensitive to temperature, light, and solvent exposure, which can lead to degradation during the bioprinting process [101,102]. Bioink formulation requires careful optimization to maintain printability, viscosity, and stability while preserving the bioactivity of plant compounds. In addition, issues of manufacturing scalability and cost must be considered. While 3D bioprinting offers unmatched precision, it remains resource-intensive, with slow production rates, high equipment costs, and stringent sterility requirements that complicate large-scale fabrication [103]. Current efforts are focused on developing standardized bioink formulations, improving printing speed, and reducing costs through automation and modular bioprinting platforms [104]. Addressing these hurdles is essential for the clinical translation of phytochemical-loaded 3D bioprinted scaffolds from laboratory prototypes to widely accessible wound therapies.

### 6.3. Hydrogel Formulation

Hydrogel has been highly regarded as a wound treatment material due to its excellent biocompatibility, moisture retention capacity, and ability to provide a sustained release system for the delivery of drugs or other bioactive materials for therapeutic enhancement [105]. Its ability to retain water makes it not only very elastic but also flexible, thus it can be engineered to conform to the wounded area. Hydrogels can effectively absorb wound exudates on the wound surface, which helps stimulate migration of keratinocytes and proliferation of fibroblasts [106]. Since hydrogel frameworks are made of almost 90% water, they can maintain a moist environment around the wound area, which is required to accelerate tissue repair [107]. The application of hydrogel dressing has been widely utilized in burn injuries, surgical wounds, skin trauma, and pressure ulcers [108].

Hydrogel can be broadly classified as a natural polymer hydrogel and a synthetic polymer hydrogel. Natural polymer hydrogels such as alginate, chitosan, gelatin, and hyaluronic acid offer biocompatibility and biodegradability, with gradual degradation that minimizes secondary damage during dressing replacement [105]. Meanwhile, synthetic polymers such as poly(vinyl alcohol) (PVA), polyethylene glycol (PEG), and polyacrylic acid (PAA) provide greater control over physicochemical properties, as they can be precisely engineered for strength, porosity, and degradation rates. Their standardized manufacturing processes also enhance reproducibility and stability, ensuring consistent therapeutic outcomes.

The stability and performance of hydrogels are often tailored through crosslinking methods. Hydrogel can be differentiated according to the types of cross-linking, either physically or chemically. Chemically crosslinked hydrogels possess high mechanical strength due to permanent covalent bonds but require careful removal of potentially toxic crosslinking agents before clinical use [109]. In contrast, physically crosslinked hydrogels rely on reversible interactions such as hydrogen bonding and hydrophobic forces, which make them responsive to environmental stimuli like pH and temperature. These mild gelation conditions are particularly attractive for incorporating plant-derived compounds, many of which are heat- or solvent-sensitive [110]. Such responsiveness can also be harnessed to design “smart” wound dressings capable of releasing bioactives in response to the wound microenvironment.

Recent studies highlight the therapeutic potential of plant bioactive-loaded hydrogels. For example, encapsulation of *Theobroma cacao* extract loaded hydrogel using carbopol-940 via the dispersion method has shown sustained compound release with improved anti-microbial and antioxidant effects for cutaneous wound healing [111]. Additionally, a hybrid approach using alginate/gelatin hydrogel incorporated with nanosilver and plant extracts (plantain peel, *A. vera*, *C. officinalis*, and curcumin) has shown superior effects in terms of antibacterial activity, cell viability, and wound closure in comparison with the hydrogel with nanosilver alone for wound treatment [112]. Similarly, in another study, a Gelatin Methacryloyl (GelMA) based hydrogel formulated with *K. ericoides* leaf extracts (KELE@Gel) was developed as a versatile dressing for wound application. The formulation exhibited excellent cytocompatibility and ROS scavenging activity in vitro and enhanced re-epithelialization, angiogenesis, collagen migration, antioxidant, anti-inflammatory, and immunoregulatory properties in the in vivo diabetic wound model [113].

Despite these advantages, processing challenges remain a barrier to translation. Many phytochemicals are unstable under high temperatures or in the presence of harsh solvents, which can compromise their bioactivity during hydrogel preparation [101]. In addition, controlling release kinetics while maintaining hydrogel structural integrity can be complex, particularly when combining multiple bioactive agents. From a scalability and cost perspective, natural polymer hydrogels can face variability in raw material quality, while synthetic hydrogels, although consistent, may require costly purification and specialized facilities [113]. Large-scale manufacturing of hydrogel dressings with reproducible bioactive loading and release remains a challenge, as batch-to-batch consistency is critical for regulatory approval. Future work must therefore focus on optimizing fabrication techniques and scaling up cost-effective production methods without compromising therapeutic efficacy.

### 6.4. Lyophilization

Lyophilization, also known as freeze-drying, is a low-temperature dehydration technique widely used for fabricating porous scaffolds and preserving bioactive compounds. In this process, a solution is first frozen at very low temperatures (commonly −80 °C), followed by primary drying under reduced pressure that causes ice crystals to sublimate directly. A secondary drying step removes residual bound water via desorption [114]. Because of the low processing temperature, this technique is particularly suitable for incorporating temperature-sensitive phytochemicals, which remain stable while water and solvents are removed without the need for extensive rinsing. The resulting scaffolds typically possess high porosity and interconnected structures, both of which are advantageous for wound exudate absorption and nutrient diffusion.

Several studies have demonstrated the potential of lyophilization in producing plant bioactive-loaded scaffolds. For example, a pectin–alginate dressing fabricated by freeze-drying and subsequently coated with *C. oblongifolius* extract exhibited >90% porosity and an optimal moisture vapor transmission rate. The addition of the plant extract conferred antibacterial activity against *E. coli*, demonstrating its potential to reduce infection risk in wounds [115]. In another work, lyophilized alginate/carboxymethyl cellulose scaffolds loaded with *S. oleracea* extract showed good mechanical stability and biocompatibility, while the embedded phytoconstituents provided antioxidant activity to promote tissue repair [116]. Similarly, incorporation of *C. quadrangularis* extract into scaffolds via a nanoparticle-mediated approach—where double emulsion-synthesized PCL/PVA nanoparticles were blended into a chitosan–collagen–hydroxyapatite matrix before freeze-drying—resulted in a sustained release profile, controlled burst release, and well-defined pore sizes (90–100 µm), which are beneficial for cell infiltration and tissue regeneration [117].

Despite these advantages, processing challenges limit the wider clinical adoption of lyophilization for phytochemicals-loaded scaffolds for wound healing. The technique is energy-intensive, time-consuming, and requires specialized freeze-drying equipment, which increases production costs. Moreover, although lyophilization preserves thermolabile phytochemicals, certain compounds remain vulnerable to degradation due to pH or solvent compatibility issues during pre-freezing solution preparation. Controlling pore size and distribution at scale can also be difficult, leading to batch variability that affects scaffold reproducibility [118]. From a scalability perspective, large-scale production is hindered by the long drying cycles and high costs of freeze-drying, which restrict its industrial feasibility. Future research is therefore directed toward process optimization, such as reducing cycle time, incorporating cryoprotectants to stabilize phytochemicals, and developing hybrid lyophilization–3D printing strategies to improve efficiency, reproducibility, and cost-effectiveness.

## 7. Conclusion, Challenges, and Future Directions

In summary, the integration of plant-derived bioactive compounds into biomaterial scaffolds offers considerable promise for advancing wound therapy. These phytochemicals provide a diverse range of biological activities, from modulating inflammation to scavenging free radicals and reducing infection risks. Yet, their effects are not uniform, and variations in potency, stability, and mechanism of action must be recognized to avoid oversimplification. Moreover, while many compounds demonstrate compelling antioxidant and antimicrobial effects in vitro, translating these findings into clinically meaningful outcomes remains a significant hurdle due to the complexity of the wound microenvironment.

To move forward, the development of specialized scaffold systems that ensure controlled release, enhanced stability, and bioavailability of phytochemicals will be essential. Such tailored approaches may help overcome current barriers and bridge the gap between laboratory potential and clinical reality, ultimately laying the foundation for safer and more effective wound healing strategies.

## Figures and Tables

**Table 1:** Common scaffold material used in tissue engineering.

Type of Scaffold	Example	Application for Wound Healing	Advantages	Limitations	References
Synthetic Polymer	Polylactic acid (PLA), Polycaprolactone (PCL)	Wound dressings, controlled phytochemical release	Customizability in mechanical strength, degradation rate and surface characteristics	Potential toxicity, non-degradable byproduct	[77]
Natural Polymer	Chitosan, silk, alginates	Wound healing, antimicrobial and antioxidant bioactive delivery	Biocompatible, biodegradable, sustainable and versatile	Lower mechanical strength, limited scalability, variable degradation	[78,79]
Hydrogel	Agarose, alginate, collagen	Moist wound healing, controlled release of plant bioactives	Biocompatible, mimic the ECM, supports vascularization	Poor mechanical properties, difficult to sterilize	[80]
Decellularized matrices	Tissue extracellular matrix	Advanced wound dressing	Biocompatible, biomimetic, versatile, supports vascularization	Risk of immune rejection, complex production process, limited scalability	[81,82]
Ceramic	Hydroxyapatite, tricalcium phosphate, nanobioceramics	Chronic wound composite, for bone-related defects	Compressive strength, osteoconductivity, biocompatibility, enhanced tissue interaction	Brittle nature, low degradation rate, not suitable for soft tissue application	[83]

## Data Availability

The data presented in this study are available on request from the corresponding author.
